# Associations between breastfeeding duration and adherence to complementary feeding recommendations in Scotland

**DOI:** 10.1111/mcn.13633

**Published:** 2024-02-20

**Authors:** Ada L. Garcia, Jiali Huang, Charlotte M. Wright

**Affiliations:** ^1^ Human Nutrition, School of Medicine, Dentistry & Nursing University of Glasgow Glasgow UK

**Keywords:** breastfeeding, child nutrition, commercial baby foods, complementary feeding, infant diet

## Abstract

We aimed to describe how breastfeeding relates to adherence to complementary feeding (CF) recommendations, diet diversification and feeding skills development and whether sociodemographic factors explain any differences observed. The Scottish Maternal Infant and Nutrition Survey for infants aged 8–12 months collected breastfeeding history, CF practices, diet and sociodemographic data using a self‐completion questionnaire. Non‐healthful CF practices were starting CF < 6 months, any consumption of sugar‐sweetened beverages (SSBs), sweet or salty snacks (treats) or unmodified cow's milk and regular consumption of commercial baby foods. Diet diversification and feeding skills were assessed by amount of self‐feeding and number of food groups, meals and snacks eaten daily. Of the 2730 mothers, 20% were solely infant formula fed (IFF) and 48% continued breastfeeding ≥6 months. Compared to IFF babies, mothers who gave any breast milk ≥6 months were more likely to start CF ≥ 6 months compared to those IFF (66% vs. 37%) and less likely to give treats (15% vs. 45%), SSBs (11% vs. 20%) and commercial baby foods (31% vs. 53%). These associations remained highly significant (*p* < 0.001) even after sociodemographic factor adjustment. Despite starting CF later, infants breastfed ≥6 months ate the same number of food groups and meals as those IFF, were just as likely to self‐feed purees and more likely to self‐feed finger foods daily (87% vs. 81% *p* < 0.001). Mothers who breastfeed beyond 6 months adhere more to CF recommendations and start CF later compared to IFF, but their babies eat a similarly diverse diet and have similar feeding skills.

## Key messages

- Adhering to breastfeeding recommendations is associated with delayed introduction of complementary foods, without influencing diet diversity or development of infant feeding skills.
- Breastfeeding duration is a predictor for healthful complementary feeding practices.
- These associations between breastfeeding duration and healthful complementary feeding practices are not explained by differences in maternal age or deprivation level.

## Graphical abstract

Breastfeeding for more than 6 months is related to healthier complementary feeding practices. Compared to formula‐fed babies, babies fed any breast milk for longer are more likely to start complementary feeding at 6 months, eat less treats and commercial baby foods, drink less sugar‐sweetened beverages and feed themselves finger foods. Maternal age or socioeconomic status did not explain these relationships.

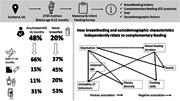

## 1 INTRODUCTION

Infant feeding recommendations emphasise the crucial role of breastfeeding for the first 6 months to support the infant's optimal growth and reduce the risk of infection (Pérez‐Escamilla et al., 2023). The transition to complementary feeding (CF) from 6 months is needed to meet nutrient demands via diet diversification, but also for the development of feeding skills and to establish healthful eating habits (WHO, 2023). Parents are recommended to introduce suitable family foods, progressing from purees to more textured meals and finger‐size foods and offer green, bitter vegetables. They are also advised to avoid adding salt and sugar to foods and avoid exposure to sweet flavours, in particular, ‘treats’ such as chocolate or confectionery (SACN, 2018). Commercial baby foods are not encouraged because they are mostly sweet, and have limited texture, while baby snacks are nutritionally inadequate and may encourage future snacking behaviour (Garcia et al., 2020). Recommended liquids are breast milk, infant formula or plain water, instead of flavoured sweet drinks (Fewtrell et al., 2017; SACN, 2018). Adherence to infant feeding recommendations in the UK has improved over the past decades for age of CF, and quality of foods (Spyreli et al., 2022). Nevertheless, global concerns exist on the role of commercial determinants of food choice in early years (Baker et al., 2023) and the expansion and pervasive nature of marketing by the baby food industry (Garcia et al., 2022; Ghisolfi et al., 2013; Hawkes et al., 2015).

Both exposure to breastfeeding and age of first CF are frequently described in observational studies as important predictors of later diet and growth. Breastfeeding has been associated with healthier diets in late childhood (Burnier et al., 2011; Collins et al., 2016; Fonseca et al., 2019; Moss et al., 2020; Spaniol et al., 2020). On the other hand, exclusively breastfed babies start complementary foods later, which has been hypothesised as potentially resulting in poorer CF skills and delayed diet diversification (Coulthard et al., 2009; Nicklaus, 2011). However, infant feeding choices do not occur in isolation and a challenge in observational studies is to allow for the role of sociodemographic confounding factors that are well‐established predictors of milk feeding choices and adherence to feeding recommendations (Dubois & Girard, 2003; Scott et al., 2009).

The Scottish Maternal and Infant Nutrition Survey provides detailed information on milk and CF from a representative survey of mothers with infants aged 8–12 months (Scottish Government, 2017) that allows an exploration of how different key predictors of CF interact and mediate each other. We aimed to investigate (1) the inter‐relationship of mode of milk feeding with the extent to which mothers were following healthful infant feeding recommendations, (2) the association of breastfeeding with development of feeding skills, diet variety and diversification of taste and texture in the diet and (3) the extent to which these associations are explained by sociodemographic factors.

## 2 METHODS

### 2.1 Study design

The Scottish Maternal and Infant Nutrition Survey 2017, a population survey commissioned by the Scottish Government, comprised cross‐sectional samples of three different groups (Scottish Government, 2017). Data from the third survey of mothers of infants aged 8–12 months were used in this study. Between March and July 2017, all 9246 mothers who gave birth in Scotland between July and August 2016 were mailed survey packs and 2730 (30%) of those who confirmed having a baby aged 8–12 months and returned a questionnaire.

### 2.2 Milk and CF practices

The survey included questions on when breast milk was last given, if ever, and current and previous infant formula use. CF practices recorded were age of first complementary foods, and frequency of consumption (every day, 5–6 day/week, 2–4 day/week, once/week, <once week, never) of commercial baby foods (e.g., pouches, snacks, jars, tubs, bars), and diet variety by frequency of consumption of the following foods: breakfast cereals or porridge, any type of potatoes, lentils, beans, sweet potatoes, rice or pasta, any type of bread, eggs, meat or fish, whole or pureed fruit, green leafy vegetables, any other vegetables not including potatoes and dairy produce but not including milk.

Other questions related to feeding practices were average number of meals per day (excluding drinks, snacks and treats), consumption of drinks other than breast milk or infant formula (cow's milk, other type of milk, water, fresh fruit juice, sugar‐free drinks, sugar‐sweetened beverages [SSBs], tea, coffee, fizzy drinks), ‘treats’ (including chocolate, ice cream or savoury snack foods such as crisps) and other snacks (not including milk or treats).

Development of feeding skills was measured using questions on the extent to which children fed themselves spoonable purees or finger foods (e.g., carrot sticks, slices of apple, cubes of cheese).

### 2.3 Sociodemographic data

Sociodemographic data included maternal age, ethnicity and the Scottish Index of Multiple Deprivation (SIMD), a proxy of deprivation based on postal codes covering aspects such as health, education, access to services, income, employment, access to services, crime and housing, with SIMD 1 indicating most deprived to SIMD 5 indicating least deprived.

### 2.4 Statistical analysis

To test associations between breastfeeding and CF practices, binary logistic regression was used, with milk feeding as a categorical predictor variable (never breast fed, feeding any breast milk <6 months and continuing feeding any breast milk >6 months) with unhealthful/not recommended feeding practices as binary outcomes. These were categorised as first CF before 6 months, any consumption of SSBs, any sweet or salty snack foods, any unmodified cow's milk and regular consumption of commercial baby foods (on 5 or more days per week). In adjusted models, deprivation (SIMD) and maternal age were also included in the model as ordinal variables.

To test associations between milk feeding and feeding skills, a similar logistic regression model was used, but with breastfeeding entered as an ordinal variable, none = 0, <6m = 1 and >6m = 2, with the outcome being whether the child self‐fed meals or snacks at least daily, again adjusted for deprivation (SIMD) child age and maternal age.

The associations between milk feeding and diversification of taste/texture were tested using multiple linear regression models, with the predictors being breastfeeding category, SIMD, child age and maternal age and the continuous outcomes being daily number of meals and snacks (including treats) and number of food groups eaten on most days (diet variety).

The association of SIMD and maternal age with feeding skills was tested using the *χ* ^2^ trend.

### 2.5 Ethical statement

The survey was approved by the Public Benefit and Privacy Panel for Health and Social Care and the National Health Service (NHS) Scotland in December 2016 (Scottish Government, 2017).

## 3 RESULTS

There were 2730 mothers who completed the survey when their babies were aged 7–12 months (4.6% 6–7 months, 58% 8 months, 31% 9 months and 6.6% 10–11 months); the great majority were White Scottish or British (80%), with only 5% of South Asian origin and 12% other white European. Only 26% of mothers were aged <30 years, 35% were between 30 and 35 years and 37% were 35 or above. Deprivation was relatively underrepresented for the most deprived SIMD quintiles (29% in SIMD 1 or 2) compared to 53% living in the most affluent (SIMD 4 or 5). Half of the mothers were primiparous. Overall, a high proportion had breast fed at least partially, with only 20% solely IFF from birth and 48% continuing till beyond 6 months; both SIMD and maternal age were strongly related to ever breastfeeding (*p* < 0.001) (Table 1).

**Table 1:** Association of sociodemographic factors with breastfeeding and complementary feeding practices.

Sociodemographic variables	Number	Any breastfeeding	Self‐fed purees<daily	Self‐fed finger foods<daily	Number of meals,	Number of snacks (including treats)	Number of food groups most days
		% (*N*)	% (*N*)	% (*N*)	Mean (SD)	Mean (SD)	Mean (SD)
All maternal age	2730	80.2% (2189)	69.5% (1725)	16.5% (416)	2.91 (0.49)	1.42 (1.1)	5.00 (1.8)
<30	707	69.6% (492)	66.0% (422)	19.2% (123)	2.88 (0.56)	1.63 (1.4)	4.74 (1.9)
30–34	1014	82.9 (841)	70.5% (650)	15.5% (145)	2.93 (0.46)	1.21 (1.1)	5.15 (1.8)
35+	1009	84.8 (856)	70.8% (653)	15.7% (148)	2.92 (0.46)	1.28 (1.1)	5.04 (1.8)
*p χ* ^2^ trend		<0.001	0.06	0.09	0.329	<0.001	<0.003
Scottish Index of Multiple Deprivation							
1 Most deprived	345	71.3 (246)	73.3% (214)	24.7% (74)	2.82 (0.64)	1.73 (1.3)	4.45 (1.9)
2	444	69.4 (308)	69.2% (265)	18.9% (76)	2.88 (0.53)	1.56 (1.2)	4.77 (1.8)
3	540	79.4 (429)	64.3% (322)	15.1% (77)	2.94 (0.42)	1.44 (1.1)	5.02 (1.8)
4	670	83.6 (645)	70.4% (439)	13.6% (84)	2.93 (0.47)	1.33 (1.1)	5.15 (1.8)
5 Least deprived	730	88.4 (645)	71.0% (485)	15.2% (105)	2.94 (0.43)	1.25 (1.1)	5.27 (1.7)
*p χ* ^2^ trend		<0.001	0.16	0.004	<0.001	<0.001	<0.001

Only 17.5% started CF before 5 months, 35% started between 5 and 6 months and 47% waited till 6 months. Recommendations were generally well observed, but 26% babies ate treats daily and 14% had SSB drinks, though only 4% had unmodified cow's milk. Commercial baby foods were used by 73%, with 39% giving them most days or every day.

Most babies (83%) were reported to feed themselves finger foods at least daily, but only 30% had begun feeding themselves purees. Out of nine possible food groups, only 50% of children ate foods from three or more groups daily, while 25% ate two or fewer. However, on most days, 50% ate five or more food groups, but 9% ate two or less. Most children (82%) had three meals per day, with the median number of all eating occasions per day being 4, but 16% ate 6–12 times per day in total, reflecting a wide range of snack frequencies, with 24% having no regular snack, but 16% having three or more snacks.

Deprivation predicted both breastfeeding duration and the extent to which mothers were following healthful dietary recommendations; mothers in the most deprived quintile were four times more likely to be giving SSBs (26%) than those in the most affluent mothers (6.4%) and twice as likely to be giving treats (39% vs. 18%). Regular use of commercial baby foods differed less, but was also more common (46% vs. 36%). All these differences were significant, *p* < 0.001. Maternal age was also relatively predictive of feeding practices. Mothers aged <30 years were more likely to start CF before 6 months (59%) than mothers aged >35 (46%) and were also more likely to give SSBs (22% vs. 12%) and treats (37% vs. 21%). All these differences were also significant, *p* < 0.001, but younger mothers were not more likely to use commercial baby foods (43% vs. 40%).

Greater affluence (low SIMD) was also associated with increased self‐feeding of finger foods, number of meals and number of food groups and with reduced snacking. Babies of younger mothers had more snacks and ate fewer food groups but were more likely to self‐feed purees (Table 1).

### 3.1 Association of breastfeeding with CF practices

There was a strong graded relationship between exposure to breastfeeding and adherence to all recommendations, except for giving milk, and these associations remained highly significant, even after adjustment for child and maternal age and SIMD, although the associations with SSBs and treats were moderately attenuated by adjustment (Table 2).

**Table 2:** Association of breastfeeding with complementary feeding practices.

Breastfeeding	Started complementary feeding <6 m	Any treats	Any SSBs	Regular baby foods	Other milk
Never	62.7% (336)	44.8% (239)	19.8% (107)	53.4% (288)	4.4% (24)
<6 months	60.0% (518)	29.7% (256)	15.4% (133)	42.8% (370)	4.5% (39)
>6 months	44.2% (582)	15.4% (202)	11.1% (147)	30.9% (405)	3.6% (47)
*p Χ* ^2^	<0.001	<0.001	<0.001	<0.001	0.29
Odds ratio^a^ (95% CI interval)					
Never	Ref	Ref	Ref	Ref	Ref
<6 months	0.89 (0.7–1.1)	0.52 (0.4–0.7)	0.65 (0.5–0.8)	0.74 (0.5–0.98)	1.02 (0.6–1.7)
>6 months	0.47 (0.4–0.6)	0.23 (0.2–0.3)	0.39 (0.3–0.5)	0.51 (0.4–0.7)	0.80 (0.5– 1.3)
Adjusted odds ratio^b^ (95% CI interval)					
Never	Ref	Ref	Ref	Ref	Ref
<6 months	0.92 (0.7–1.2)	0.56 (0.4–0.7)	0.83 (0.6–1.1)	0.66 (0.5–0.8)	1.1 (0.6–1.8)
>6 months	0.51 (0.4–0.6)	0.27 (0.2–0.3)	0.64 (0.5–0.8)	0.39 (0.3–0.5)	0.93 (0.5–1.5)

*Note*: Breastfeeding and complementary feeding practices are reported as % (*N*).

^a^ Binary logistic regression with breastfeeding as the sole categorical predictor.

^b^ Binary logistic regression with breastfeeding as the categorical predictor, plus maternal age and SIMD.

### 3.2 Association of breastfeeding duration with diet diversity, development of feeding skills and diversification of tastes and textures

Longer breastfeeding duration was associated with more self‐finger feeding but not with self‐feeding purees or the number of meals offered. In univariate analysis, breastfed children ate more food groups than those IFF, but this difference became nonsignificant after adjustment for SIMD and child age. Breastfeeding duration was significantly related to lower snack consumption, even after adjustment (Table 3).

**Table 3:** Association of breastfeeding with diet diversity, feeding skills and diversification of taste and texture.

Breastfeeding	Never, % (*N*)	<6 months, % (*N*)	>6 months, % (N)	*p χ* ^2^	Odds ratio (OR)^a^ (95% CI), *p* value	Adjusted OR^b^ (95% CI), *p* value	Other predictors in the adjusted model
Self‐fed purees daily	32.1% (152)	28.3% (226)	31.4% (380)	NS	1.01 (0.9–1.1), 0.88	1.01 (0.9–1.3), 0.89	Child age
Self‐fed finger foods daily	81.3% (400)	80.2% (647)	86.6% (1056)	*p* < 0.001	1.26 (1.1–1.4), <0.001	1.20 (1.05–1.4), 0.008	Child age, SIMD

	Mean (SD)	Mean (SD)	Mean (SD)	*p* ANOVA	Βeta^c^ (95% CI), *p* value	Βeta^c^ (95% CI), *p* value	
Number of meals	2.9 (0.46)	2.9 (0.48)	2.9 (0.49)	0.26	−0.022 (−0.04 to 0.01), 0.26	‐	‐
Number of snacks (incl treats)	1.3 (0.87)	1.1 (0.85)	1.0 (0.87)	<0.001	−0.20 (−0.3 to −0.2), <0.001	−0.18 (−0.3 to −0.2), <0.001	Child age, SIMD, maternal age
Number of food groups consumed most days^d^	4.78 (1.9)	5.11 (1.8)	5.03 (1.8)	0.036	0.04 (0.01–0.2), 0.036	0.022 (−0.4 to 0.1), 0.25	Child age, SIMD

Abbreviation: CI, confidence interval.

^a^ Odds ratio binary logistic regression with self‐feed purees and finger foods as outcomes and breastfeeding as the only predictor, entered as an ordinal variable (none = 0, <6m = 1 and >6m = 2).

^b^ Odds ratio logistic regression for self‐feed purees and finger foods as outcomes, with breastfeeding plus other significant variables as predictors.

^c^ Standardised regression coefficient for number of meals, snacks, and food groups consumed most days as outcomes, with breastfeeding as a predictor.

^d^ Standardised regression coefficient for number of meals, snacks, and food groups consumed most days with breastfeeding plus other significant variables as predictors.

## 4 DISCUSSION

Exclusivity and duration of breastfeeding are known to be associated with healthier eating patterns in later childhood. Evidence from longitudinal observational studies in later childhood has shown that exclusive breastfeeding for 3–6 months predicts higher fruit and vegetable intake (Burnier et al., 2011; Moss et al., 2020), reduced consumption of ultra‐processed foods (Fonseca et al., 2019) and lower intake of SSBs (Spaniol et al., 2020). Less evidence is available for earlier years, but a cohort study in Australia has described breastfeeding as a predictor of higher intake of healthier foods during the first 3 years of life (Collins et al., 2016). We thus set out to investigate whether the amount of exposure to breast milk during the first 6 months of life was associated with CF practices at age 8–12 months and the extent to which any differences could be explained by differences in the sociodemographic characteristics of breastfeeding compared to infant formula‐feeding mothers.

In this population survey in Scotland, adhering to infant feeding recommendations was fairly common, with 50%–80% following different recommendations; this is encouraging in terms of public health efforts to promote optimal nutrition in early years. Similar adherence to CF feeding recommendations has also been reported in a smaller UK online survey with 400 participants mostly residing in England, but with a large proportion of mothers highly educated and with high income (Spyreli et al., 2022).

This may reflect general improvements in feeding practices reported elsewhere; for example, breastfeeding uptake at age 6–8 weeks in Scotland has increased in the last decade from 36% to 45%, which is encouraging, though still far from universal coverage (Public Health Scotland, &, 2021). Meanwhile, substantial progress has been made for age of complementary food introduction over the past two decades, with 79% in 2021 waiting until around 6 months (Public Health Scotland, &, 2021) compared to 50% giving complementary food by age 4 months in 2000 (Alder et al., 2004).

Even though progress has been made, diet inequalities persist. In this study, infants in more deprived households were more likely to be fed treats and SSBs and offered less diet variety compared to those less deprived. This compounds the risk of lower exposure to breast milk in children in more deprived areas, illustrated by recent routine Scottish data that show a 30% point gap for any breastfeeding at 6–8 weeks between the most deprived (30%) and the least deprived (62%) areas (Public Health Scotland, &, 2021). While the provision of healthful CF is strongly socially patterned, this does not seem to mediate its association with the length of time mothers breast feed. This suggests that those who persist with breastfeeding have a wider commitment to healthful feeding that does not simply reflect their underlying levels of deprivation or culture. The development of feeding skills and diversification of tastes was less strongly and consistently socially patterned and only some aspects related to breastfeeding duration.

A theoretical concern is that if most breastfeeding infants start CF later, they will be delayed in acquiring feeding skills and diversifying their diet. It is thus reassuring that infants breast fed for the longest still ate the same number of food groups and meals as IFF babies, were just as likely to self‐feed purees and were more likely to self‐feed finger foods daily. Trial evidence has shown that longer breastfeeding duration as per current recommendations has no impact on children's acceptance of complementary foods during CF (Cohen et al., 1995); indeed, children who breast fed closer to the 6 months' recommendation ate a wider variety of foods during CF, including consuming more finger foods such as fruit and vegetables than those receiving infant formula (Taylor et al., 2017). Furthermore, findings from a cohort study in Australia showed that prolonged breastfeeding was a strong predictor of higher food variety, including consuming more fruit and vegetables at 2 years of age (Scott et al., 2012).

Our findings, although of an observational nature, build a case to support the concept of ‘healthful infant feeding trajectories’ where breastfeeding sets the foundation to healthful eating practices away from market‐driven food environments that are harmful (Pérez‐Escamilla et al., 2023; Rollins et al., 2023), particularly for those most socioeconomically vulnerable. Further interventional research is needed to consolidate this concept.

### 4.1 Strengths and limitations

A strength of the study is the population‐based approach. The sampling design was representative, inviting mothers of all babies born in the relevant time period to join the study. However, in common with most surveys of this nature (McAndrew et al., 2012), the response rate was only 30% and the breastfeeding rates in this survey are much higher than those reported in routinely collected data, in particular, areas of high deprivation (Public Health Scotland, &, 2021). This suggests some response bias, with participants self‐selected to participate who are more interested in feeding and might be more educated, as described in other surveys (Spyreli et al., 2022). However, while there was a lower than expected proportion of participants living in more deprived households, there was still substantial representation from these more deprived SIMD strata, making it possible to examine and allow for sociodemographic effects. It is unfortunate that no information was collected on maternal education, as this is a strong predictor of infant feeding practices (Collins et al., 2016), as well as health‐related outcomes such as growth or developmental status.

Data on diet‐related exposure in this age group in the UK or similar setting are scarce and hard to obtain. While there is no detailed dietary assessment, the survey still provides rich data on types of complementary foods consumed as well as feeding styles.

With data collection carried out in 2017, these are the most up‐to‐date representative data available in this population group, until the much‐needed reinstatement of infant feeding surveillance in the UK.

## 5 CONCLUSIONS

In this cross‐sectional population survey in Scotland, mothers of infants breast fed beyond 6 months had more healthful CF practices than mothers who IFF, independent of socioeconomic status and maternal age. Breastfeeding infants start CF later compared to IFF babies but eat a similarly diverse diet and have similar feeding skills.

## AUTHOR CONTRIBUTIONS

Ada L. Garcia conceptualised the study; Jiali Huang analysed data and contributed to the initial paper draft; Charlotte M. Wright analysed data and contributed to conceptualisation. Ada L. Garcia wrote the paper and was responsible for the paper's final content. All authors read and approved the final manuscript.

## CONFLICT OF INTEREST STATEMENT

The authors declare no conflict of interest.

## Data Availability

The data that support the findings of this study are openly available in The UK National Archives at https://www.nationalarchives.gov.uk/doc/open-government-licence/version/3/.
